# A Hybrid Network for Large-Scale Action Recognition from RGB and Depth Modalities

**DOI:** 10.3390/s20113305

**Published:** 2020-06-10

**Authors:** Huogen Wang, Zhanjie Song, Wanqing Li, Pichao Wang

**Affiliations:** 1 School of Electrical and Information Engineering, Tianjin University, Tianjin 300072, China; 2 Advanced Multimedia Research Lab, University of Wollongong, NSW 2522, Australia; wanqing@uow.edu.au; 3 School of Mathematics, Tianjin University, Tianjin 300350, China; zhanjiesong@tju.edu.cn; 4 Alibaba Group (U.S.) Inc., Bellevue, WA 98004, USA; pw212@uowmail.edu.au

**Keywords:** action recognition, weighted rank pooling, weighted dynamic image, 3D convolutional LSTM network, canonical correlation analysis

## Abstract

The paper presents a novel hybrid network for large-scale action recognition from multiple modalities. The network is built upon the proposed weighted dynamic images. It effectively leverages the strengths of the emerging Convolutional Neural Network (CNN) and Recurrent Neural Network (RNN) based approaches to specifically address the challenges that occur in large-scale action recognition and are not fully dealt with by the state-of-the-art methods. Specifically, the proposed hybrid network consists of a CNN based component and an RNN based component. Features extracted by the two components are fused through canonical correlation analysis and then fed to a linear Support Vector Machine (SVM) for classification. The proposed network achieved state-of-the-art results on the ChaLearn LAP IsoGD, NTU RGB+D and Multi-modal & Multi-view & Interactive ($M^{2}I$) datasets and outperformed existing methods by a large margin (over 10 percentage points in some cases).

## 1. Introduction

Recognition of human actions from RGB-D data has attracted increasing attention over the past years due to the fast development of easy-to-use and cost-effective RGB-D sensors such as Microsoft Kinect, Asus Xtion, and recently Intel’s RealSense. These RGB-D sensors capture RGB video together with depth sequences. The RGB modality provides appearance information whereas the depth modality, being insensitive to illumination variations, provides 3D geometric information. Skeletons can also be extracted from either depth maps [1] or RGB video [2] under certain conditions, for instance, the subjects being in a standing position and not being overly occluded. As the seminal work [3], research on action recognition [4] from RGB-D data has extensively focused on using either skeletons [5,6] or depth maps [7], some work using multiple modalities including RGB video. However, single modality alone often fails to recognize some actions, such as human-object interactions, that require both 3D geometric and appearance information to characterize the body movement and the objects being interacted. Unlike most existing multimodality action recognition methods [4] using skeletons plus depth or RGB-video, this paper presents a novel and deep neural network-based method to recognize actions from RGB video and depth maps.

Throughout the research in recent years, four promising deep neural network approaches to action recognition have emerged. They are two-stream convolutional neural networks (CNNs) [8], 3D CNNs [9,10], CNNs, either 2D or 3D, combined with a recurrent neural network (RNN) [11] and dynamic image (DI) based approaches [12]. The two-stream CNN approach captures spatial and temporal information by two parallel streams, one being dedicated for appearance and the other for motion. The 3D CNN approach employs 3D convolutions and 3D pooling operations to learn spatiotemporal information over video segments. Both two-stream CNN and 3D CNN approaches obtained the video level prediction by averaging predictions over sampled segments, the long-term temporal information was not explicitly considered. The CNN plus RNN approach extracts spatial features from frames or short segments of frames using CNNs and feeds the spatial features to an RNN, commonly LSTM, to exploit temporal information. This architecture often tends to over-emphasize the temporal information. Dynamic image-based methods encode action instances into one or more dynamic images and employ existing CNN models directly on the dynamic images for classification. Dynamic images are good at capturing spatial information including pose and interactions with objects but tend to lose some temporal information.

As observed, each of the four approaches has its own strength and weakness in capturing and utilizing spatial, temporal and structural information required for robust action recognition. To a large extent, this explains why none of the individual approaches would perform robustly on a large-scale action dataset (e.g., ChaLearn LAP IsoGD Dataset [13], NTU RGB-D Dataset [14]) where discriminative power or importance of the spatial, temporal and structural information [4] varies significantly from action to action. Considering as attributes the relative duration of discriminative motion in an action instance and involvement of interactions with objects, the types of actions in the popular RGB-D action datasets [14] can be broadly categorized into four groups:

C1  The discriminative motion distributes throughout an action and there is no interaction with objects. Typical examples are “stand up” and “sit down”.
C2  The discriminative motion appears only in a short period within the duration of an action and there is no interaction with objects. Examples are “nodding” and “cough”.
C3  The discriminative motion distributes throughout an action and it involves interaction with objects. Examples are “put on the jacket” and “take off jacket”.
C4  The discriminative motion appears only a short period within the duration of an action and there is interaction with objects. Typical examples include “eat meals/snack” and “drink water”.

Strong temporal modelling is needed to recognize actions in C1; spatial information is important for C2; structural information becomes important for C3; C4 needs the spatial information including the object pose. Table 1 shows an empirical observation on how each of the four approaches performed on the four categories of actions, and the performance of the four approaches on the four categories of actions were evaluated in the NUT RGB-D action dataset using depth modality and cross-subject protocol. Notice that the CNN+RNN (ConvLSTM) performed well for actions in the first three groups as expected due to its ability to model temporal information throughout the actions. The poor performance for actions in C4 means its inability of capturing both spatial and temporal information in a short period over the entire instance of the actions.

This paper presents a novel hybrid network that takes the advantages of the four approaches. Furthermore, the conventional dynamic images (DI) are extended to weighted dynamic images (WDI) through the proposed weighted rank pooling. Unlike conventional DIs, a WDI can account for both spatial and temporal importance adaptively and, hence, improve its performance on actions in C4 as well as other groups. A 3D ConvLSTM is constructed where 3-D convolution [9,10] is used to learn short-term spatiotemporal features from the input video, and then ConvLSTM [15] is utilized to extract long-term spatiotemporal features. Both WDI and 3D ConvLSTM are applied to RGB video and depth maps to extract features. These features are fused together using Canonical Correlation Analysis (CCA) [16,17] into an instance feature for classification. The proposed hybrid network is evaluated and verified on the ChaLearn LAP IsoGD Dataset [13], NTU RGB+D Dataset [14] and Multi-modal, Multi-view and Interactive($M^{2}I$) Dataset [18]. This paper is an extension of the conference paper [19]. The extension includes WDI, feature level fusion using Canonical Correlation Analysis, a detailed justification of the proposed network, additional experiments on the NTU RGB+D Dataset and $M^{2}I$ Dataset and comparison with the methods reported recently.

The remainder of this paper is organised as follows. Section 2 reviews the related work on deep learning-based action recognition and fusion methods. Section 3 describes the proposed weighted rank pooling method. Section 4 presents the details of the hybrid network. Section 5 presents the experimental results and discussions. The paper is concluded in Section 6.

## 2. Related Work

This section presents a review of the related works including the major deep-learning-based approaches and the fusion methods commonly used in multiple modalities based action recognition.

### 2.1. Deep Learning-Based Action Recognition

Much work has been reported on action recognition from RGB-D sequences based on deep learning. The four emerging and promising approaches are Two-stream CNNs [8], 3D CNN [9,10], CNNs combined with an RNN [11] and Dynamic Image (DI)-based methods [12].

#### 2.1.1. Two-Stream CNNs

The two-stream architecture [8] employs two CNNs to learn appearance and motion features from RGB frames and stacked optical flow, respectively. Then several mechanisms were proposed in [20,21] to fuse the two networks. According to the observation that discriminative information may be sparsely distributed in a few segments within a video and most other segments are redundant for the action labelled, a key segment deep mining framework is designed in [22] to search key video segments and perform classification simultaneously. To incorporate long-range temporal information, Wang et al. proposed a temporal segment network that sparsely samples frames from a video sequence during training and classification scores of the sampled frames are aggregated to a final one in testing [23]. However, frames are processed independently. Their experiments have shown that the performance seems to be independent of the number of sampled frames, which indicates that the network may have failed to capture long-range temporal information. In addition, the extraction of optical flow is a resource-demanding process though optical flow can be replaced by motion vectors directly extracted from compressed videos [24] or extracted by MotionNet [25]. In general, the two-stream CNN architecture captures spatial information and short-term temporal information but hardly learns long-term temporal information.

#### 2.1.2. 3D CNN

A 3D CNN extends a 2D CNN, both in convolution and pooling, to the temporal domain. It was first proposed in [9] with 3D kernels. Later, an architecture named as C3D is presented in [10] to extract spatiotemporal features. 3D CNNs has become an effective tool for action recognition. However, the performance of 3D CNNs failed to overcome the one of two-stream CNNs. To overcome the failure, Carreira and Zisserman achieved a great breakthrough using the inflation of 2D kernels pretrained on ImageNet into 3D ones [26]. However, 3D CNNs increase both memory usage and complexity due to the increasing number of parameters of the spatiotemporal filters. Several different strategies were introduced to mitigate these drawbacks. One can decompose a 3D convolutional kernel into 2D spatial convolution and 1D temporal convolution [27,28,29,30]. One can also integrate 2D CNNs with the 3D convolution module to generate deeper and more informative feature maps [31,32]. However, each 3D convolution usually covers small temporal windows rather than the entire video, so they can only encode short temporal information. Like the two-stream CNN approach, the 3D CNN approach captures spatial information and short-term temporal information, but not much long-term temporal information.

#### 2.1.3. CNN Plus RNN

This approach tackles the action recognition problem by a cascade of CNN and RNN. Donahue et al. proposed a Long-term Recurrent Convolutional Network (LRCN) [11] through a cascade of CNNs with an LSTM, in which the LSTM combines the frame-level features extracted by 2D CNNs to model spatiotemporal relationship. To weight highly relevant spatial-temporal locations or the important frames, Sharma et al. [33] extended LRCN with a soft attention model. Even though LSTM is highly capable of modelling temporal dependence, it fails to learn the intuitive high-level spatiotemporal structure. Jain et al. mined the spatio-temporal-structural information by combining spatiotemporal graphs and an RNN in [34]. However, it cannot well capture motion dynamics between the frames and the spatial correlation at the same time by directly applying LSTM to video-based action recognition. Since ConvLSTM [15] only considers neighbouring pixels’ relationship in the spatial domain, Zhu et al. [35] adopted 3D CNN and ConvLSTM to extend the spatial neighbour to temporal neighbour for gesture recognition from the depth and RGB modalities. Sun et al. proposed a Lattice LSTM network [36] by extending LSTM with independent memory cell transition between RGB and optical flow streams. This method models long-term features without obviously increasing the complexity of the model and, hence, strengthens the ability to model motion dynamics across time in an effective way.

#### 2.1.4. Dynamic Image-Based Approach

This approach turns a video sequence into one or multiple dynamic images (DIs) that aim to encode both spatial and temporal information, and then applies a CNN to classify the dynamic images. Bilen et al. [12] proposed to adopt rank pooling [37] to convert a video sequence into one set of dynamic images and use them to fine-tune the models pre-trained on ImageNet [38]. Fernando et al. proposed the end-to-end learning methods with rank pooling for learning discriminative representations of videos [39]. To improve the DI’s ability to encode long-term temporal dependency, a hierarchical rank pooling scheme that encodes a video sequence at multiple levels was proposed in [40]. This method divides a video sequence into multiple overlapping video segments and encodes each video segment using rank pooling to produce a sequence of DIs. The resulting DI sequence also is divided into multiple subsequences and rank pooling is applied to each of these subsequences. By recursively applying rank pooling on the obtained segment DIs from the previous layer, high-order complex dynamics are expected to be captured. Although a dynamic image is effective for summarizing a video sequence, it cannot always capture the properties required to identify the video because the ranking constraints are linear. Cherian et al. introduced generalized rank pooling to overcome these drawbacks with the quadratic ranking function [41]. However, it does not consider the fact that the importance of the order between any two frames and pixels or region in each frame would vary from action to action. In this paper, we propose a weighted dynamic image to overcome this limitation.

In general, different action categories do not benefit equally from the spatial, temporal and structural information. But current action recognition methods do not take into account this property and cannot adaptively exploit the spatial, temporal and structural information. We address this problem by proposing a new novel hybrid architecture built upon the proposed weighted dynamic images and a cascade of 3D convolution and ConvLSTM. This architecture has led to state-of-the-art performance on the popular and large-scale datasets.

### 2.2. Fusion Methods

The commonly used method to fuse multiple modalities for action recognition is either score fusion or feature concatenation. Score fusion combines prediction scores from the classifiers independently trained on individual modalities through maximum, average or multiplication operations. Simonyan et al. utilized score fusion to combine the softmax scores of two independent CNNs [8] in the two-stream CNN method. Wang et al. used average score fusion to combine the classification scores obtained from multiple weighted hierarchical depth motion maps [42]. Although the score fusion is effective in many cases, the differences among the contributions to the classification by individual modalities were not considered in these methods. On the other hand, feature concatenation often integrates features before classification by direct concatenation of the features extracted from individual modalities. Yu et al. concatenated semantic features, long-term temporal features, and short-term temporal features of a video [43]. Ji et al. concatenated object features, motion features and scene features from videos for linear classification [44]. However, the fundamental assumption of feature concatenation is that features are independent of and complementary to each other. Simple concatenation does not remove potential hidden redundancy among features that could lead to an adverse effect on the classification. Such redundancy inevitably exists among the features from different modalities, especially when they are extracted independently. In addition, the curse of dimensionality may occur when the number of modalities increases and reduction of dimensionality is essentially required. In this paper, we adopt CCA to fuse the features extracted from different modalities and reduce the dimensionality at the same time. The fused features are then fed to a linear SVM for action recognition. Compared with score fusion, the CCA-based feature fusion achieved much better performance as demonstrated in the experiments.

## 3. Proposed Weighted Rank Pooling

Rank pooling [45] is usually used to capture sequence-wide temporal evolution. However, conventional rank pooling often ignores the fact that frames in a sequence are of different importance and regions in frames also are of different importance to the classification [46]. As discussed in Section 1, different frames in an action instance contribute differently to the recognition and some frames contain more discriminative information than others. In addition, a frame can be decomposed into salient and non-salient regions [47]. Compared with non-salient regions, salient regions contain information of the discriminative foreground. To accommodate both frame-based and region-based importance, spatial weights and temporal weights are proposed to be integrated into the rank pooling process, referred to as weighted rank pooling. In the rest of this section, we first give a general formulation of the proposed weighted rank pooling and then discuss the two types of weights.

### 3.1. Formulation

Given a sequence *X* of *n* feature vectors, $X=<x_{1},x_{2},\cdots ,x_{n}>$, where $x_{i}\in R^{D}$ is the feature of frame *i*. Each of the elements $x_{i}$ may be a frame itself or the feature extracted from the frame. Spatial weight $V=<v_{1},v_{2},\cdots \;v_{n}>$ represents the importance of each element of the features in frames and $v_{i}\in R^{D}$. The temporal weight $W=<w_{1},w_{2},\cdots ,w_{n}>$ indicates the importance of the frames in the sequence and $w_{i}\in R$. In this paper, it is assumed that $\sum_{i=1}^{n}w_{i}=1$.

Based on the frame representations $x_{i}$, we define a memory map ψ over the time variable *i*, $\psi :i\to \psi_{i}$ where $\psi_{i}\in R^{D}$. The output of the vector-valued function $\psi_{i}$ is obtained by processing all the frames up to time *i*, denoted by $\psi_{i}$. In this paper, we define $\psi_{i}$ as:

(1) $$\psi_{i}=\frac{\underset{t=1}{\sum^{i}}v_{t}*x_{t}}{i},$$

here * is Hadamard Product.

Rank pooling focuses on relative ordering (i.e., $\psi_{i+1}$ succeeds $\psi_{i}$ which forms an ordering denoted by $\psi_{i+1}≻\psi_{i}$). Frames are ranked based on $\psi_{i}\left(i=1,\cdots ,n\right)$. A natural way to model such order constraints is a pairwise linear ranking machine. The ranking machine learns a linear function characterized by the parameters $u\in R^{D}$, namely $\phi \left(\psi ;u\right)=u^{T}\cdot \psi$. The ranking score of $\psi_{i}$ is obtained by $\phi \left(\psi_{i},u\right)=u^{T}\cdot \psi_{i}$ and results in the pairwise constraints ($\psi_{i+1}≻\psi_{i}$). The learning to rank problem optimizes the parameters *u* of the function ϕ(ψ,u), such that $\psi_{j}≻\psi_{i}\Leftrightarrow \phi \left(\psi_{j},u\right)>\phi \left(\psi_{i},u\right)$. We argue that the importance of the ordering of each pair of frames in an instance of action should be different and dependent on the category of the action. Therefore, we propose to use ω(i,j) as a weighting factor denoting the importance of the ordering of frames *i* and *j*.

The process of weighted rank pooling is to find $u^{*}$ to minimize the following objective function:

(2) $$\underset{u}{min}\frac{1}{2}{∥u∥}^{2}+C\sum_{\forall i,j,i>j}\varepsilon_{ij},s.t.\;\forall i,j,i>j:\omega \left(i,j\right)\left(u^{T}\cdot \psi_{i}-u^{T}\cdot \psi_{j}\right)\geq 1-\varepsilon_{ij},$$

here *i* and *j* are the indices of frames in the sequence. $\varepsilon_{ij}>0$ is a threshold enforcing the temporal order and *C* is a regularization constant. A pairwise function ω(i,j) computes a scalar representing the importance of the order between frame *i* and frame *j*. The pairwise function ω(i,j) can be measured by the temporal weight $w_{i}$ and $w_{j}$. In this paper, ω(i,j) is represented by $\omega \left(i,j\right)=max\left(w_{i},w_{j}\right)$ though many other forms of the function are also feasible. As the ranking function $\phi (\psi_{i},u)$ is sequence specific, the parameters *u* would capture a sequence-wide spatially and temporally weighted representation and can be used as a descriptor of the sequence.

### 3.2. Optimization

Equation (2) aims to find *u* by minimizing the number of pairs of frames in the training examples that are switched their desired order. We obtain *u* by solving the following optimization problem:

(3) $$\begin{array}{cc} \; & \underset{u}{min}\frac{1}{2}{∥u∥}^{2}+C\sum_{\forall i,j,i>j}max{\left(0,1-\omega \left(i,j\right)\left(u^{T}\cdot \psi_{i}-u^{T}\cdot \psi_{j}\right)\right)}^{2} \end{array}$$

Equation (3) can be solved efficiently in many ways as described in [48]. As it is an unconstrained and differentiable objective function, Truncated Newton optimization is adopted, in which the parameter *u* can be updated at each iteration as Equation (4).

(4) $$\begin{array}{c} u\leftarrow u-H^{-1}g \end{array}$$

where *g* is the gradient of the objective function, *H* is the Hessian of the objective function. The gradient of Equation (3) is,

(5) $$\begin{array}{cc} \; & g:=u+2C\sum_{\forall i,j\in sv}\omega \left(i,j\right)\left(\omega \left(i,j\right)u^{T}\left(\psi_{i}-\psi_{j}\right)-1\right)\left(\psi_{i}-\psi_{j}\right) \end{array}$$

and its Hessian is

(6) $$\begin{array}{cc} \; & H:=I+2C\sum_{\forall i,j\in sv}\omega^{2}\left(i,j\right)\left(\psi_{i}-\psi_{j}\right){\left(\psi_{i}-\psi_{j}\right)}^{T} \end{array}$$

$H^{-1}g$ can be calculated with linear conjugate gradient through the matrix-vector multiplication Hs for a vector *s*. If we assign $q_{k}\in Q=\left\{\omega \left(i,j\right)|i>j\right\}$, Hs can be computed as follows:

(7) $$\begin{array}{cc} \; & Hs=s+2CQ^{T}D\left(Qs\right) \end{array}$$

where *D* is a diagonal matrix with $D_{kk}=1$ if $u^{T}q_{k}<1$; 0 otherwise. Detailed steps to solve Equation (3) is shown in Algorithm 1.

**Algorithm 1**: The solution of Equation (3). The Newton step is computed with linear conjugate gradient.**Input**: $S=(<\left(x_{1},1\right),\cdots ,\left(x_{n},n\right)>,V,W,C)$
$\psi_{i}=\frac{\underset{t=1}{\sum^{i}}v_{t}*x_{t}}{i}$

$\omega \left(i,j\right)=max\left(w_{i},w_{j}\right)$

$q_{k}\in Q=\left\{\omega \left(i,j\right)|i>j\right\}$
*u* is randomly iniatlized
**repeat**
 $D_{kk}=1\left(u^{T}q_{k}<1\right)$ $g=u+2CQ^{T}D\left(Q^{T}u-1\right)$ **repeat**  Update based on the computation of $s+2CQ^{T}D\left(Qs\right)$ for some *s*. **until** Convergence of linear conjugate gradient $\delta u={\left(I+2CQ^{T}DQ\right)}^{-1}g$ u←u−τδu (τ found by line gradient)**until** Convergence of Newton
**return**
*u*


### 3.3. Discussion

In this section, we discuss several possible ways to compute the spatial weight $v_{i}$ and the temporal weight w(i) in the proposed weighted rank pooling. Learning of the weights is possible, but is beyond the scope of the paper.

#### 3.3.1. Spatial Weights

The spatial weight $v_{i}$ indicates the importance of each spatial location in frame *i*. When the location *p* in frame *i* is important, $v_{i}\left(p\right)$ is assigned to a large value, otherwise, $v_{i}\left(p\right)$ is assigned to a small value. The spatial weights can be estimated by a spatial attention model, background-foreground segmentation, salient region detection, or flow-guided aggregation.

#### 3.3.2. Temporal Weights

The temporal weight $w_{i}$ indicates the importance of each frame in a sequence. $w_{i}$ is a scalar. When the frame *i* is important, $w_{i}$ is assigned to a large real number, otherwise, a smaller real number is assigned. The temporal weights could be estimated by a temporal attention model, selection of key frames, or flow-guided frame weights.

#### 3.3.3. Weighted Rank Pooling vs. Rank Pooling

If the spatial weight $v_{i}$ is a unit matrix and the temporal weight w(i) equals to $\frac{1}{n}$, the proposed weighted rank pooling is equivalent to rank pooling. In other words, conventional rank pooling is a special case of the proposed weighted rank pooling.

### 3.4. Bidirectional Weighted Rank Pooling

The weighted rank pooling ranks the accumulated feature $\psi_{i}$ up to the current time *t*, thus the pooled feature is likely biased towards the early frames and subject to the order of frames. However, future frames beyond *t* are also usually useful to classify frame *t*. To use all available input frames, the weighted rank pooling can be applied in a bidirectional way to convert one video sequence into a forward dynamic image and a backward dynamic image.

## 4. Proposed Hybrid Network Architecture

**Figure 1:**
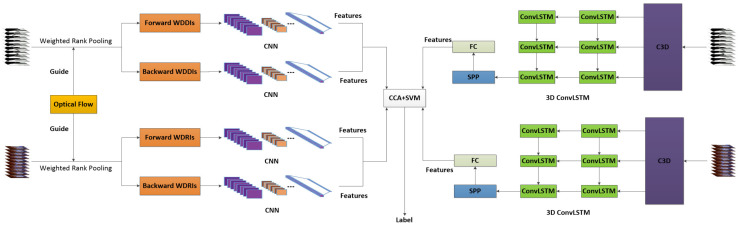
An overview of the proposed hybrid network for multimodal action recognition. The network is built upon the proposed weighted dynamic images, CNNs and 3D ConvLSTM to extract highly complementary information from the depth and RGB video sequences. Canonical correlation analysis is adopted for feature-level fusion and a linear SVM for classification.

This section presents the proposed hybrid network architecture and its key components. As shown in Figure 1, the proposed network consists of three types of components: CNN-based component that takes weighted dynamic images as input, 3D ConvLSTMs based component that takes as input video and depth sequences and the multi-stream fusion component that fuses the outputs from the CNNs and 3D ConvLSTMs for final action recognition. Weighted dynamic images are constructed from both RGB and depth sequences and fed into CNNs to extract features. At the same time, the RGB and depth sequences are input to the 3D ConvLSTMs to extract features. A canonical correlation analysis based fusion scheme is then applied to fuse the features learned from the CNNs and 3D ConvLSTMs, and the fused features are fed into a linear SVM for action classification.

### 4.1. CNN-Based Component

Two sets of weighted dynamic images, Weighted Dynamic Depth Images (WDDIs) and Weighted Dynamic RGB Images (WDRIs), are constructed, respectively, from depth sequences and RGB sequences through bidirectional weighted rank pooling. Given a pair of RGB and depth video sequences, the proposed bidirectional weighted rank pooling method is applied at the pixel-level to generate four weighted dynamic images, namely forward WDDI, backward WDDI, forward WDRI and backward WDRI. Specifically, in this paper, spatial and temporal weights in the weighed rank pooling are calculated from optical-flows, where the average flow magnitude of a frame is considered as temporal weight of the frame and the flow magnitude of each pixel is treated as the spatial weight of that pixel.

**Figure 2:**
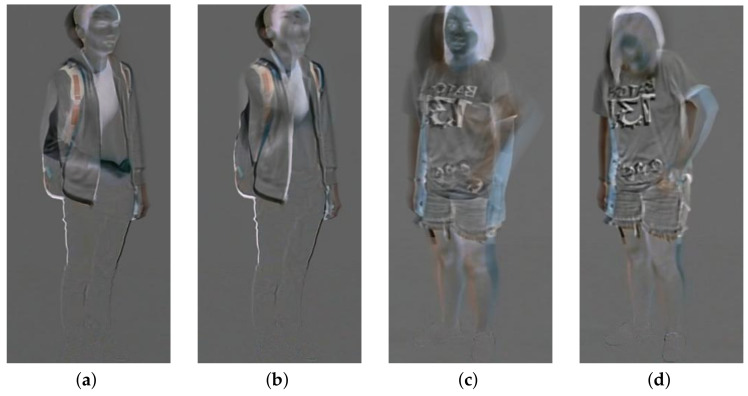
The dynamic images of action from the NTU RGB + D Dataset. (**a**) a conventional dynamic image of “eating meal/snack”; (**b**) a weighted dynamic image of “eating meal/snack”; (**c**) a conventional dynamic image of “put something inside pocket / take out something from pocket”; (**d**) a weighted dynamic image of “put something inside pocket / take out something from pocket”.

Different from the conventional rank pooling, weighted rank pooling can capture more effectively the discriminative spatiotemporal information. As shown in Figure 2, the conventional dynamic image of action “eat meal/snack” from the NTU RGB+D Dataset [14] does not capture the process of putting things into the mouth whereas the weighted dynamic image of “eat meal/snack” presents the discriminative part of eating. The hand motion around the pocket is suppressed by the head and body motion in the conventional dynamic image of action “put something inside pocket/take out something from pocket” from the NTU RGB+D Dataset, but the hand motion around pocket is encoded in the weighted dynamic image. Four ConvNets were trained on the four channels individually, forward WDDI, backward WDDI, forward WDRI and backward WDRI. ResNet-50 [49] is adopted as the CNN model in this paper through other CNN models are also applicable. The details of ResNet-50 can be found in [49]. The learned features from last pooling layer of the ResNets are named respectively as $S_{FD}$, $S_{BD}$, $S_{FR}$ and $S_{BR}$.

### 4.2. 3D ConvLSTM Based Component

The 3D ConvLSTM presented in Zhu et al. [35] is adopted to learn spatiotemporal information of actions. In particular, a 3-D convolution network is to extract short-term spatio-temporal features and the features are then fed to a ConvLSTM to model long-term temporal dynamics. Finally, the spatiotemporal features are normalized with Spatial Pyramid Pooling (SPP) [50] for the final classification. The details of 3D ConvLSTM can refer to [35]. In the proposed hybrid architecture, both RGB and depth sequences are processed independently in two streams. This part of the proposed hybrid network leverages the strengths of the conventional two-stream CNN and CNN+RNN approaches. The features extracted from the SPP layer on the RGB stream and depth stream are denoted as $T_{R}$ and $T_{D}$, respectively.

### 4.3. CCA Based Feature Fusion

Considering the potential correlation between features extracted from the RGB video and depth maps by the CNNs and 3D ConvLSTMs, the simple and traditional feature concatenation is not effective as such concatenation would lead to information redundancy and high dimensionality of the fused features. Therefore, we adopt a canonical correlation analysis (CCA) [16,17] to remove redundancy across the features and fuse them. CCA fusion can keep effective discriminant information and reduce the dimension of the fused features at the same time.

Given two heterogeneous feature vectors $X\in R^{p\times n}$ and $Y\in R^{q\times n}$ containing *n* samples of different features. Their covariance matrix of $\left(\begin{array}{c} X\\ Y \end{array}\right)$ is denoted as

(8) $$S=\left(\begin{array}{cc} S_{xx} & S_{xy}\\ S_{yx} & S_{yy} \end{array}\right)$$

where $S_{xy}\in R^{p\times q}$ is the covariance matrix between *X* and *Y* ($S_{xy}^{T}=S_{yx}$), and $S_{xx}\in R^{p\times p}$ and $S_{yy}\in R^{q\times q}$ are the within-set covariance matrices of *X* and *Y*. CCA aims to find a pair of canonical variables with $X^{*}=W_{x}^{T}X$ and $Y^{*}=W_{y}^{T}Y$ to maximize the correlation across two feature sets. The goal of the CCA is to maximize the following objective function.

(9) $$Corr\left(X^{*},Y^{*}\right)=\frac{cov(X^{*},Y^{*})}{\sqrt{var\left(X^{*}\right)*var\left(Y^{*}\right)}}$$

where $cov\left(X^{*},Y^{*}\right)=W_{x}^{T}S_{xy}W_{y}$, $var\left(X^{*}\right)=W_{x}^{T}S_{xx}W_{x}$ and $var\left(Y^{*}\right)=W_{y}^{T}S_{yy}W_{y}$. Because the problem in (9) is invariant with scaling of $W_{x}$ and $W_{y}$, the objective function is reformulated as follows:

(10) $$argmax\;W_{x}^{T}S_{xy}W_{y}\;s.t.\;W_{x}^{T}S_{xx}W_{x}=1,W_{y}^{T}S_{yy}W_{y}=1$$

We can use SVD to solve the optimization problem. The variance matrices $S_{xx}$ and $S_{yy}$ are firstly transformed into identity forms.

(11) $$S_{xx}=S_{xx}^{1/2}S_{xx}^{1/2}\;and\;S_{yy}=S_{yy}^{1/2}S_{yy}^{1/2}$$

Applying the inverses of the square root factors symmetrically on the joint covariance matrix in Equation (8) we obtain

(12) $$\begin{array}{c} \left(\begin{array}{cc} S_{xx}^{-1/2} & 0\\ 0 & S_{yy}^{-1/2} \end{array}\right)\left(\begin{array}{cc} S_{xx} & S_{xy}\\ S_{yx} & S_{yy} \end{array}\right)\left(\begin{array}{cc} S_{xx}^{-1/2} & 0\\ 0 & S_{yy}^{-1/2} \end{array}\right)\\ =\left(\begin{array}{cc} I_{q} & S_{xx}^{-1/2}S_{xy}S_{yy}^{-1/2}\\ S_{yy}^{-1/2}S_{yx}S_{xx}^{-1/2} & I_{p} \end{array}\right) \end{array}$$

$W_{x}$ and $W_{y}$ can be obtained by solving the SVD:

(13) $$S_{xx}^{-1/2}S_{xy}S_{yy}^{-1/2}=U^{T}SV$$

where the columns of *U* and *V* correspond to the sets of orthonormal left and right singular vectors, respectively. The singular values of matrix *S* correspond to the canonical correlations.$W_{x}$ and $W_{y}$ can be given by

(14) $$W_{x}=S_{xx}^{-1/2}U\;W_{y}=S_{yy}^{-1/2}V$$

Finally, the fused feature *Z* is obtained as follows.

(15) $$Z=W_{x}^{T}X+W_{y}^{T}Y={\left(\begin{array}{c} W_{x}\\ W_{y} \end{array}\right)}^{T}\left(\begin{array}{c} X\\ Y \end{array}\right)$$

In this paper, $S_{FD}$ and $S_{BD}$, $S_{FR}$ and $S_{BR}$ are firstly fused into $S_{D}$ and $S_{R}$ by CCA fusion, respectively. Then $S_{D}$ and $T_{D}$, $S_{R}$ and $T_{R}$ are fused into $Z_{D}$ and $Z_{R}$, respectively. Finally, $Z_{D}$ and $Z_{R}$ combined into *Z* by CCA fusion. In this paper, $Z\in R^{512\times n}$, where *n* is the number of samples, and 512 is the dimension of the feature. A linear SVM classifier is trained on the fused feature *Z* for final action recognition.

## 5. Experiments

The proposed network was evaluated on ChaLearn LAP IsoGD Dataset [13], NTU RGB + D Dataset [14], and Multi-modal & Multi-view & Interactive($M^{2}I$) Dataset [18]. These datasets cover a variety of actions including gestures, daily living activities and interactions.

### 5.1. Network Training

#### 5.1.1. Training of the CNNs

ResNet-50 [49] was adopted as the CNN model. For the ChaLearn LAP IsoGD Dataset, we fine-tuned the CNNs on the Forward WDDIs with the pre-trained model on ImageNet [38], and then fine-tuned separately the CNNs on the Backward WDDIs and Forward WDRIs with the trained model on the Forward WDDIs. Finally, we fine-tuned the CNNs on the Backward WDRIs with the trained model on the Forward WDRIs. The networks were fine-tuned for both NTU RGB + D Dataset and $M^{2}I$ Dataset based on the trained models on the ChaLearn LAP IsoGD Dataset. The network was trained using mini-batch stochastic gradient descent with the momentum being set to 0.9 and the weight decay being set to 0.0001. The batch size is 16. The activation function used in all hidden weight layers is RELU. With respect to data augmentation, horizontal flipping and corner cropping were used. The learning rate for fine-tuning was set to $10^{-4}$, and then it was decreased to its 0.96 every 40K iterations. The maximum number of iterations is set to 90,000. The TVL1 optical flow algorithm [51] implemented in OpenCV with CUDA was used to extract the optical flow. The CNNs were implemented with Caffe [52] and trained on one TITAN X Pascal GPU.

#### 5.1.2. Training of the 3D ConvLSTM Network

The 3D ConvLSTM network was implemented with the Tensorflow [53] and Tensorlayer platforms and trained on one TITAN X Pascal GPU. Given a pair of RGB and depth video sequences, RGB and depth modalities are fed into the two separately 3D ConvLSTM networks. Since no pre-trained models are available for the 3D ConvLSTM networks, we first trained the network on the depth modality of the ChaLearn LAP IsoGD Dataset from scratch. Then, we fine-tuned the RGB based network based on the pre-trained model of the depth modality. The networks were fine-tuned for both NTU RGB + D Dataset and $M^{2}I$ Dataset based on the models trained on the ChaLearn LAP IsoGD Dataset. The initial learning rate was set to 0.1 and decreased to its 1/10 every 15K iterations. The weight decay was initialized as 0.004 and decreased to 0.00004 after 40K iterations. The maximum number of iterations is set to 60K. At each iteration, the batch-size is 13, the temporal length of each clip is 32 frames, and the crop size for each image is 112×112.

### 5.2. Evaluation of Different Settings and Comparision

#### 5.2.1. Weighted Dynamic Images vs. Dynamic Images

Table 2 compares the performance using Weighted Dynamic Images including Weighted Dynamic Depth Images (WDDIs) and Weighted Dynamic RGB Images (WDRIs), and Dynamic Images including Dynamic Depth Images (DDIs), Dynamic RGB Images (DRIs) on the validation set of ChaLearn LAP IsoGD Dataset. From the results, we can see that Weighted Dynamic Images improved the performance by 6.5 percentage points over Dynamic Images. Notice that the Weighted Dynamic RGB Images also outperforms Dynamic Images by 9.37 percentage points. This verifies that the proposed Weighted Dynamic Images are more robust and more discriminative.

#### 5.2.2. Evaluation of Different Spatial/Temporal Weights Estimation Method

In this section, we take the depth modality in ChaLearn LAP IsoGD Dataset as an example to evaluate the different spatial/temporal weights estimation methods. The results are shown in Table 3. The first group is the result of a convenient dynamic image. The second group is the results of different spatial weights estimation method, and the last group is the results of different temporal weights estimation method.

For the spatial weight estimation method, we compared the results of background-foreground segmentation, salient region detection, and flow-guided aggregation. For background-foreground segmentation, a nonparametric background model, the most reliable background model (MRBM) [54], was adopted to the segment foreground area. The model can relate the best estimate of the background to the modes (local maxima) of the underlying distribution and model the variation of the background. The spatial weight $v_{i}$ is assigned to 1 when the pixel is in the foreground area, and the spatial weight $v_{i}$ is assigned to 0 when the pixel is in the background area. For salient region detection, global contrast-based salient region detection [55] was used. The spatial weight $v_{i}$ is assigned to 1 when the pixel is in salient region, and the spatial weight $v_{i}$ is assigned to 0 for other region.

For the temporal weight estimation method, the results of the selection key frames and flow-guided frame weight are listed. To select the key frame, an unsupervised learning method [56] was employed. The temporal weight $w_{i}$ is assigned to 1 for the key frames and 0 for other frames. The results show that flow-guided weighted estimation method obtains the outstanding performance in both groups.

#### 5.2.3. Features from CNNs and ConvLSTM Networks

In this section, we study whether the features extracted from the CNN component and 3D ConvLSTM component can improve the performance of each other. The action recognition performance using features extracted by the CNN component, the 3D ConvLSTM components, and the combination of them was evaluated respectively. Without losing the validity and for the sake of simplicity, average score fusion was used in this experiment. The results are summarized in Table 4.

The fusion of the recognition using the CNN features and 3D ConvLSTM futures achieved respectively 5.17 percentage point improvement (i.e., 55.67% vs. 50.50%) on the depth stream and 6.92 percentage point improvement (i.e., 55.52% vs. 48.60%) on the RGB stream compared to the best single model on the IsoGD dataset. In addition, the fusion of the recognition from all features offered 4.48 percentage point improvement on the IsoGD dataset. The fusion results on both streams have demonstrated that the CNN component and 3D ConvLSTM component of the proposed hybrid network have captured different and discriminative features. The results have verified the analysis in Section 1 and the effectiveness of the design of the proposed network.

#### 5.2.4. Feature Fusion

We evaluated the CCA based fusion scheme with a linear SVM on the IsoGD dataset. Table 5 presents the results and comparison to several popular fusion schemes. The first group shows the results of the average score fusion obtained by a linear SVM on the four individual feature channels. The results using bag-of-visual-words (BoW) [57] and Fisher Vector encoding (FV) [58] with a linear SVM are shown in the second group and the third group, respectively. The results using CCA with a linear SVM are presented in the last group. From the table, it shows that CCA based feature fusion offers performance gain compared with score-level fusion, BoW, and FV.

### 5.3. Comparison with the State-of-the-Art Results

#### 5.3.1. Results on the NTU RGB + D Dataset

NTU RGB+D Dataset is currently one of the largest RGB+D action recognition datasets, which contains 60 different action classes, and includes more than 56,000 sequences and 4,000,000 frames. The challenge of this dataset comes from the viewpoint variation and large intra-class.

The performance of our proposed method for both the cross-subject and cross-view protocols are summarized in Table 6. Firstly, we compare our method with skeleton-based approaches such as Lie Group [59], Dynamic Skeletons [60], Hierarchical recurrent neural network (HBRNN) [61], Part-aware LSTM [14], ST-LSTM + Trust Gate [62], Joint Trajectory Maps (JTM) [63], Joint Distance Maps (JDM) [64], Geometric Features [65], Clips + CNN + MTLN [66], View invariant [67], and IndRNN [68]. Our results outperform all these skeleton-based approaches for both the cross-subject and cross-view protocols. Secondly, we compare our method with Pose Estimation Maps [69] on RGB modality. Our results are better than the performance of Pose Estimation Maps by 7.66% for the cross-subject protocol and 4.33% for the cross-view protocol. Thirdly, we compare our method with some results from fusing RGB and skeleton modalities including Pose-based Attention [70] and SI-MM [71]. Although both Pose-based Attention and SI-MM borrowed the skeleton information to extract local visual features around key joints from RGB videos and optical flow videos, the performance of our method only lags behind the one of SI-MM for the cross-view protocol. Finally, some results from fusing RGB and depth modalities, such as SSSCA-SSLM [72] and Aggregation Networks [73], are listed. Our method can achieve better performance than other methods fusing RGB and depth modalities. The superior performance of our method demonstrates the effectiveness of our proposed method.

Table 7 shows the performance of the two-Streams [8], 3D CNN [10], ConvLSTM [19], DI + CNN [19], and the proposed methods on the four categories of actions in the NTU RGB + D action dataset using depth modality alone and cross-subject protocol. As expected, the proposed method outperformed all other methods not only categories C3 and C4 but also the other two categories as well. It’s worth noting that the proposed method outperformed ConvLSTM and DI + CNN by more than 12 percentage points for the actions in C4. Although the proposed method obtains outstanding performance overall, we observe that this method has relatively lower performance in actions such as “touch head“, “sneeze/cough,” “writing,” and “eating a snack.” Then a comparison between the proposed method and the popular approaches such as two-Streams [8], 3D CNN [10], ConvLSTM [19], DI+CNN [19] in these actions are made. Based on the comparison result, the proposed method achieves better performance than other approaches in these actions. The recognition of these actions remains a challenge due to objects interacted with are small or the movement is not obvious in these actions.

#### 5.3.2. Results on the ChaLearn LAP IsoGD Dataset

The ChaLearn LAP IsoGD Dataset is a large-scale isolated gesture dataset including both RGB and depth video sequences. The details of this dataset are shown in Table 8. We evaluated the proposed method on both the validation subset and testing subset.

**Figure 3:**
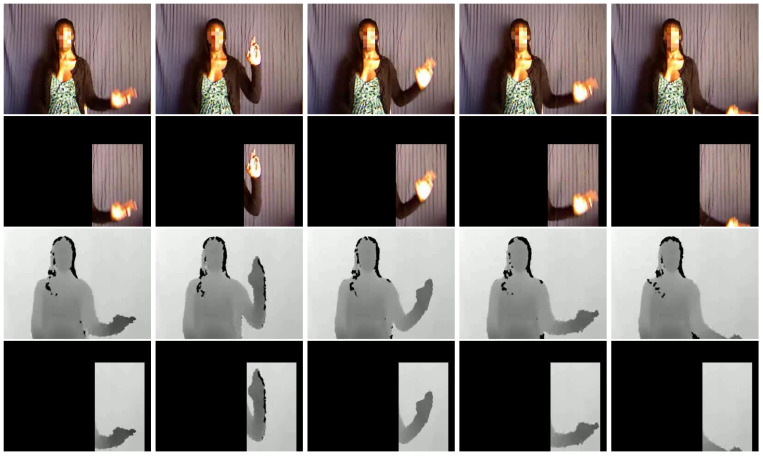
Examples of image frames at the body level and hand level. From up to bottom: body level RGB images, hand level RGB images, body level depth images and hand level depth images.

To compare with the results in [19], the proposed method was evaluated at both body level and hand level as in [19]. Gestures have both body level and hand level components. The body level component processes the whole video and looks for gross motions, while the hand level component detects and processes each hand. The body level component and the hand level component are complementary for gesture recognition. Hand regions are usually detected by color or multiple cues, but these methods are sensitive to illumination and background. Inspired by the promising performance of Faster R-CNN [74], Faster R-CNN was adopted to detect the hand regions. After the hand region detected frame by frame in a video sequence, the biggest bounding box of the hand can be detected through the whole sequence. Then the hand level images can be cropped. Examples of image frame at the body level and hand level are shown in Figure 3. Table 9 lists the performance of the proposed method at both body level and hand level, and the score fusion of body level and hand level results. The results of several methods reported in recent years are also listed in Table 9. From this Table, we can see that deep learning is more promising to extract features than hand-craft features such as MFSK [13] and MFSK + DeepID [13]. The proposed method obtains state-of-the-art performance on both validation subset and testing subset. Although 2SCVN-3DDSN [75] integrates Two Stream Consensus Voting Network (2SCVN) and 3D Depth-Saliency Network (3DDSN) and is trained on the data of four modalities (RGB, depth, optical flow, and saliency), our result is better than the performance of 2SCVN-3DDSN by 0.87% on the testing subset. These results prove the superiority of the proposed methods.

**Figure 4:**
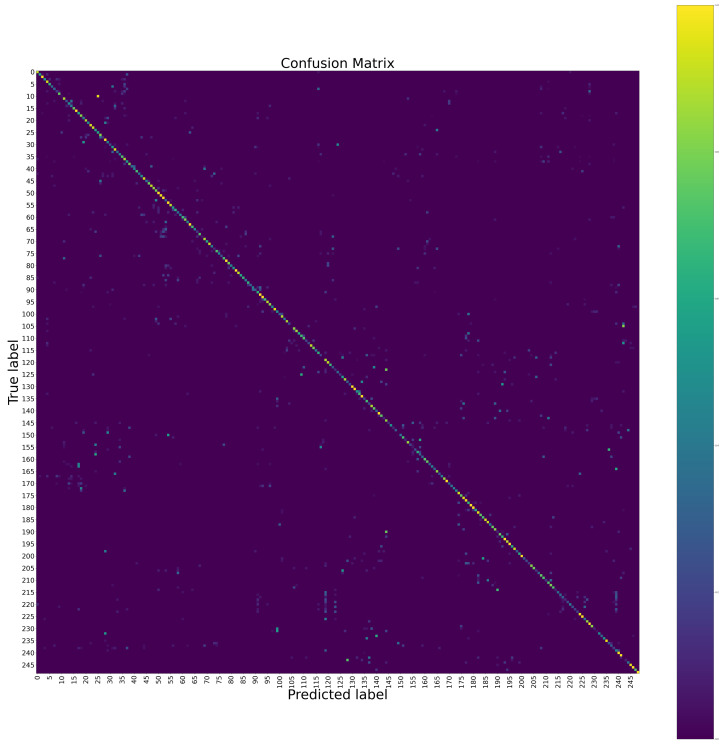
The confusion matrix of the proposed method at the hand level on the Chalearn LAP IsoGD dataset. To see the details, please zoom in.

**Figure 5:**
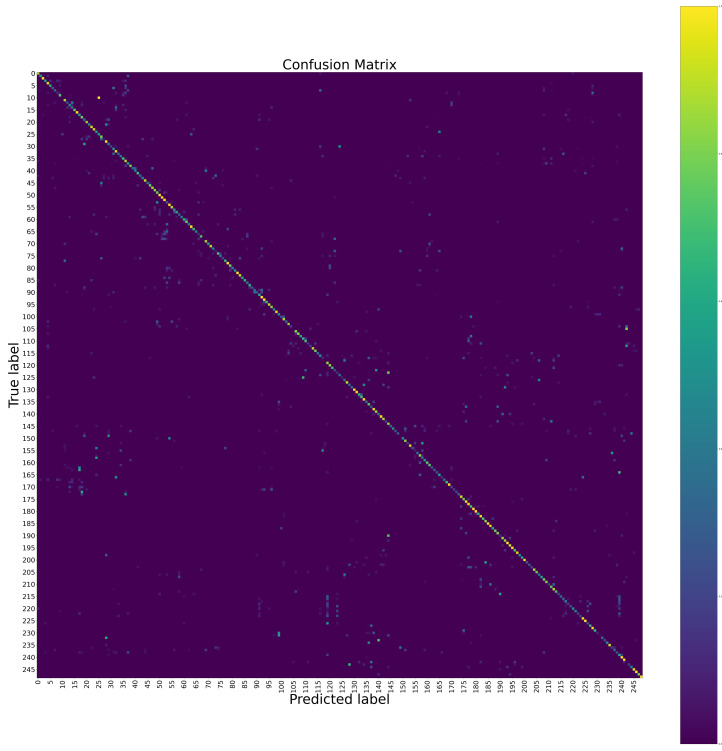
The confusion matrix of the proposed method at the body level on the Chalearn LAP IsoGD dataset. To see the details, please zoom in.

**Figure 6:**
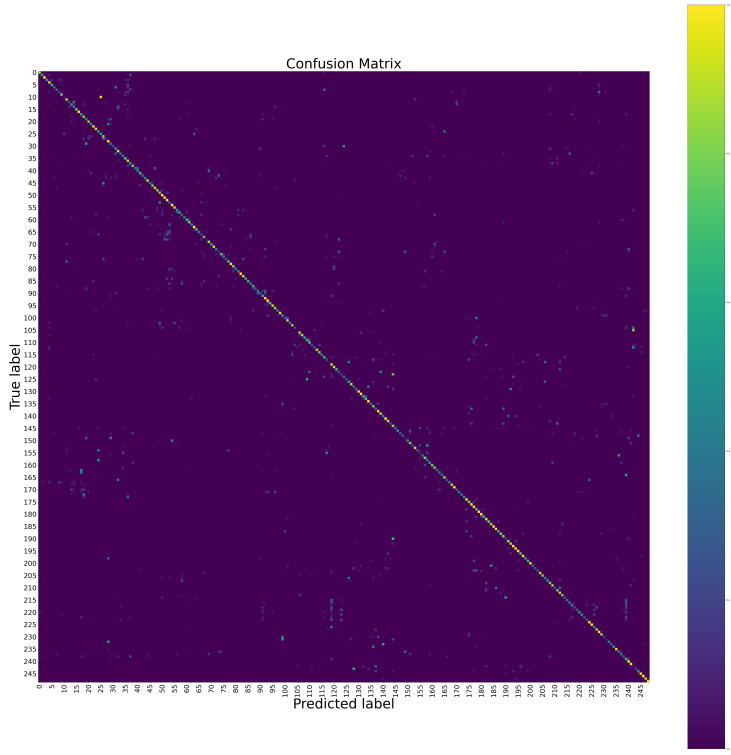
The confusion matrix of the proposed method for the fusion of the hand level and the body level on the Chalearn LAP IsoGD dataset. To see the details, please zoom in.

The confusion matrices of the proposed method at the hand level and the body level on the Chalearn LAP IsoGD dataset are shown in Figure 4 and Figure 5, respectively. The confusion matrix of the proposed method for the fusion of the hand level and the body level on the Chalearn LAP IsoGD dataset is shown in Figure 6. From these confusion matrices, we can see that the sign language like “CraneHandSignals/BoomUp” may be confused with other actions with similar motion patterns such as “CraneHandSignals/LowerLoadSlowly” at the body level, which is the weakness of using the body level image alone. The confusion matrices show that the body level component and the hand level component are complementary.

#### 5.3.3. Results on the $M^{2}I$ Dataset

$M^{2}I$ Dataset provides both human-object and human-human interactions. This dataset contains 22 action categories, and each category was performed twice by 20 groups. 8 groups are used for training, 6 groups are used for validation, and 6 groups are used for testing. The dataset is classified into Side View (SV) and Front View (FV). We followed the experimental settings in [18] and compared the results on two scenarios: single task scenario and cross-view scenario. Table 10 presents the results and comparisons on the $M^{2}I$ dataset for the single task scenario. Table 11 shows the results and comparisons for the cross-view scenario. The hand-craft methods listed in Table 10 and Table 11 such as iDT-Tra, iDT-COM, iDT-MBH, iDT-HOG + MBH, and iDT-HOG + HOF were based on iDT features [81] generated from optical flow. Although these methods are very effective in RGB based action recognition, the results in Table 10 and Table 11 shows the performance of these methods on $M^{2}I$ Dataset are limited. Compared with the deep learning methods (such as SFAM [76] and STSDDI [82]), the proposed method also achieved the best results in both scenarios. The awesome performance verify the effectiveness of the proposed method for recognizing human-object interactions and human-human interactions. This is probably because (1) the weighted dynamic images through proposed weighted rank pooling can improve the performance of recognizing human-object interactions and human-human interactions; (2) the features extracted from the CNN-based component and 3D ConvLSTM-based component can be complementary for recognizing human-object interactions and human-human interactions; (3) The pretrained model on the ChaLearn LAP IsoGD Dataset can initialize the proposed hybrid network well.

## 6. Conclusions

This paper presents an effective hybrid network for large-scale multimodal action recognition. The proposed network is built upon the proposed weighted rank pooling and takes the advantages of the 3D ConvLSTM approach. The experimental results on three popular datasets have demonstrated the efficacy of the proposed network and significant improvement of performance over the state-of-the-art methods. The proposed network can be extended to include the skeleton modality.

## Figures and Tables

**Table 1:** Performance evaluation of the two-streams, 3DCNN, CNN+RNN (ConvLSTM) and DI+CNN approaches on the NTU RGB-D action dataset using depth modality and cross-subject protocol.

Category	Two-Streams	3D CNN	ConvLSTM	DI + CNN
C1	fair (72.5%)	fair (71.8%)	good (85.5%)	good (85.0%)
C2	good (84.7%)	good (84.2%)	good (88.1%)	fair (77.0%)
C3	fair (74.1%)	poor (68.1%)	good (84.6%)	fair (71.4%)
C4	fair (73.4%)	poor (67.8%)	poor (61.8%)	fair (71.4%)

**Table 2:** Comparison of recognition accuracy using Weighted Dynamic Images and Dynamic Images on the validation set of ChaLearn LAP IsoGD Dataset.

Methods	Accuracy
DDI [19]	45.11%
DRI [19]	39.23%
Fusion of DDI and DRI [19]	49.14%
WDDI (Proposed)	50.50%
WDRI (Proposed)	48.60%
Fusion of WDDI and WDRI (Proposed)	55.64%

**Table 3:** Evaluation of different spatial/temporal weights estimation methods on the validation set of ChaLearn LAP IsoGD Dataset (only depth modality).

Methods	Accuracy
DDI	45.11%
Background-foreground segmentation	45.75%
Salient region detection	46.18%
Flow-guided aggregation	49.13%
Selection key frames	48.75%
Flow-guided frame weight	48.87%

**Table 4:** Performance on Chalearn LAP IsoGD dataset using the features extracted by the CNN and the 3D ConvLSTM components where “+” indicates average score fusion.

Modality	Feature	Accuracy
Depth	CNN	50.50%
	3D ConvLSTM	44.76%
	CNN + 3D ConvLSTM	55.67%
RGB	CNN	48.60%
	3D ConvLSTM	44.23%
	CNN + 3D ConvLSTM	55.52%
RGB + Depth	CNN + 3D ConvLSTM	60.15%

**Table 5:** Performance comparison on IsoGD with different fusion methods.

Fusion Method	IsoGD
Score Fusion (Depth)	55.67%
Score Fusion (RGB)	55.52%
Score Fusion (Depth+RGB)	60.15%
BoW+SVM (Depth)	54.91%
BoW+SVM (RGB)	55.21%
BoW+SVM (Depth+RGB)	58.93%
FV+SVM (Depth)	55.61%
FV+SVM (RGB)	55.72%
FV+SVM (Depth+RGB)	60.23%
CCA+SVM (Depth)	55.89%
CCA+SVM (RGB)	56.23%
CCA+SVM (Depth+RGB)	61.14%

**Table 6:** Comparison of the proposed method with other methods on the NTU RGB + D dataset. We report the accuracies using both the cross-subject and cross-view protocols.

Methods	Modality	Cross Subject	Cross View
Lie Group [59]	Skeleton	50.08%	52.76%
Dynamic Skeletons [60]	Skeleton	60.23%	65.22%
HBRNN [61]	Skeleton	59.07%	63.97%
Deep RNN [14]	Skeleton	56.29%	64.09%
Part-aware LSTM [14]	Skeleton	62.93%	70.27%
ST-LSTM+ Trust Gate [62]	Skeleton	69.20%	77.70%
JTM [63]	Skeleton	73.40%	75.20%
JDM [64]	Skeleton	76.20%	82.30%
Geometric Features [65]	Skeleton	70.26%	82.39%
Clips+CNN+MTLN [66]	Skeleton	79.57%	84.83%
View invariant [67]	Skeleton	80.03%	87.21%
IndRNN [68]	Skeleton	81.80%	87.97%
Pose Estimation Maps [69]	RGB	78.80%	84.21%
Pose-based Attention [70]	RGB+skeleton	82.50%	88.60%
SI-MM [71]	RGB+skeleton	85.12%	92.82%
SSSCA-SSLM [72]	RGB+Depth	74.86%	-
Aggregation Networks [73]	RGB+Depth	86.42%	89.08%
Proposed method	RGB	86.46%	88.54%
	Depth	87.73%	87.37%
	RGB+Depth	89.51%	91.68%

**Table 7:** Performance comparison of the two-streams, 3DCNN, CNN + RNN (ConvLSTM), DI + CNN methods with the proposed method on the NTU RGB + D action dataset using depth modality and cross-subject protocol.

Category	Two-Streams	3D CNN	ConvLSTM	DI + CNN	Proposed Method
C1	72.5%	71.8%	85.5%	85.0%	90.03%
C2	84.7%	84.2%	88.1%	77.2%	91.76%
C3	74.1%	68.1%	84.6%	71.4%	85.73%
C4	73.4%	67.8%	61.8%	71.4%	83.32%

**Table 8:** Information of the ChaLearn LAP IsoGD Dataset.

Sets	Gestures	RGB Videos	Depth Videos	Subjects
Training	35,878	35,878	35,878	17
Validation	5784	5784	5784	2
Testing	6271	6271	6271	2
All	47,933	47,933	47,933	21

**Table 9:** Comparison of the proposed method with other methods on the validation set and the test set of the ChaLearn LAP IsoGD.

Methods		Modality	Accuracy (Validation)	Accuracy (Testing)
MFSK [13]		RGB+Depth	18.65%	24.19%
MFSK+DeepID [13]		RGB+Depth	18.23%	23.67%
Scene Flow [76]		RGB+Depth	36.27%	-
Pyramidal C3D [77]		RGB+Depth	45.02%	50.93%
2SCVN+3DDSN [75]		RGB+Depth	49.17%	67.26%
32-frame C3D [78]		RGB+Depth	49.2%	56.9%
C3D+ConvLSTM [35]		RGB+Depth	51.02%	-
C3D+ConvLSTM+Temporal Pooling [79]		RGB+Depth	58.00%	62.14%
CNN+3D ConvLSTM [19]		RGB+Depth	60.81%	65.59%
ResC3D [80]		RGB+Depth	64.40%	67.71%
Proposed method	body level	RGB+Depth	61.14%	66.43%
	hand level	RGB+Depth	62.78%	66.23%
	Score fusion (body level + hand level)	RGB+Depth	64.61%	68.13%

**Table 10:** Comparison of the proposed method with other methods on the $M^{2}I$ dataset for the single task scenario (learning and testing in the same view).

Methods	SV	FV
iDT-Tra(BoW) [18]	69.8%	65.8%
iDT-COM(BoW)$\tilde{c}$itexu2015multi	76.9%	75.3%
iDT-COM(FV) [18]	80.7%	79.5%
iDT-MBH(BoW) [18]	77.2%	79.6%
SFAM [76]	89.4%	91.2%
STSDDI [82]	90.1%	92.1%
Proposed method	100%	100%

**Table 11:** Comparison of the proposed method with other methods on the $M^{2}I$ dataset for the cross-view scenario (SV–FV:training in the side view and test in the front view; FV–SV:training in the front view and testing in the side view).

Methods	SV–FV	FV–SV
iDT-Tra [18]	43.3%	39.2%
iDT-COM [18]	70.2%	67.7%
iDT-HOG + MBH [18]	75.8%	72.8%
iDT-HOG + HOF [18]	78.2%	72.1%
SFAM [76]	87.6%	76.5%
STSDDI [82]	86.4%	82.6%
Proposed method	93.8%	90.6%
